# The *Aedes albopictus* (Diptera: Culicidae) microbiome varies spatially and with Ascogregarine infection

**DOI:** 10.1371/journal.pntd.0008615

**Published:** 2020-08-19

**Authors:** Priscilla Seabourn, Helen Spafford, Nicole Yoneishi, Matthew Medeiros

**Affiliations:** 1 Plant and Environmental Protection Sciences, University of Hawai’i at Mānoa, Honolulu, Hawai’i, United States of America; 2 Pacific Biosciences Research Center, University of Hawai’i at Mānoa, Honolulu, Hawai’i, United States of America; Fundaçao Oswaldo Cruz, BRAZIL

## Abstract

The mosquito microbiome alters the physiological traits of medically important mosquitoes, which can scale to impact how mosquito populations sustain disease transmission. The mosquito microbiome varies significantly within individual mosquitoes and among populations, however the ecological and environmental factors that contribute to this variation are poorly understood. To further understand the factors that influence variation and diversity of the mosquito microbiome, we conducted a survey of the bacterial microbiome in the medically important mosquito, *Aedes albopictus*, on the high Pacific island of Maui, Hawai‘i. We detected three bacterial Phyla and twelve bacterial families: Proteobacteria, Acitinobacteria, and Firmicutes; and Anaplasmataceae, Acetobacteraceae, Enterobacteriaceae, Burkholderiaceae, Xanthobacteraceae, Pseudomonadaceae, Streptomycetaceae, Staphylococcaceae, Xanthomonadaceae, Beijerinckiaceae, Rhizobiaceae, and Sphingomonadaceae. The *Ae*. *albopictus* bacterial microbiota varied among geographic locations, but temperature and rainfall were uncorrelated with this spatial variation. Infection status with an ampicomplexan pathosymbiont *Ascogregarina taiwanensis* was significantly associated with the composition of the *Ae*. *albopictus* bacteriome. The bacteriomes of mosquitoes with an *A*. *taiwanensis* infection were more likely to include several bacterial symbionts, including the most abundant lineage of *Wolbachia* sp. Other symbionts like *Asaia* sp. and several Enterobacteriaceae lineages were less prevalent in *A*. *taiwanensis-*infected mosquitoes. This highlights the possibility that inter- and intra-domain interactions may structure the *Ae*. *albopictus* microbiome.

## Author summary

The microbiome is defined as a community of microorganisms (bacteria, archaea, fungi, protozoa, and viruses) living on or within a host organism. The microbiome influences physiological traits of medically important mosquitoes and can alter disease transmission dynamics in vector populations. The composition of the mosquito microbiome varies across mosquito populations; however, the factors that contribute to this variation are poorly understood. Understanding the factors that shape the mosquito microbiome will inform how mosquito-borne disease transmission varies among environments and help to develop effective disease-mitigating strategies. In this study, we assessed the diversity and variation of the microbiome of a medically important mosquito, *Aedes albopictus*. We found that the mosquito microbiome composition varies across geographic locations but is not affected by rainfall or temperature. We discovered that mosquitoes infected with the parasite *Ascogregarina taiwanensis* had a different microbiome composition than that of mosquitoes with little to no infection. This study contributes to our understanding of the factors that influence the diversity and variation in the mosquito microbiome. This and other studies like it will contribute to the development of new and innovative strategies to prevent and mitigate diseases vectored by mosquitoes.

## Introduction

A symbiotic microbiome comprises communities of microorganisms that modulate their host’s organismal function by influencing several physiological traits [1]. Complex host-microbiome interactions can shape immunity [2], nutrition, and metabolism [3, 4] and alter host susceptibility to pathogens [5]. For example, the symbionts *Hamiltonella defensa* and *Serratia symbiotica* have been shown to confer protection against parasitic wasps in aphids [6, 7], indicating a symbiont-mediated resistance against parasitism, and highlighting the intimate relationships between symbionts and their host that was shaped over evolutionary history.

In vectors, such as mosquitoes (Diptera: Culicidae), microbiome modulation of host organismal function scales to influence the capacity of vector populations to transmit pathogens [8]. Several vector traits that shape vectorial capacity are influenced directly and indirectly by the microbiome [9]. For instance, certain microbial symbionts may enhance susceptibility of *Anopheles* sp. mosquitoes to *Plasmodium* sp. parasites, while endosymbionts like *Wolbachia* sp. decrease susceptibility of *Aedes* sp. and *Anopheles* sp. mosquitoes to arboviruses and *Plasmodium* sp., respectively [10–12]. This change in vector competence might be associated with competition between microbial symbionts for resources, the release of anti-pathogen peptides that directly limit pathogen growth and extend the incubation period, or through the regulation of specific components of the insect immune system [8, 13].

The mosquito microbiome varies significantly between and within host species [14, 15]. Mosquitoes acquire their microbial symbionts from the environment across all stages of development and via vertical transmission of endosymbionts early in embryogenesis [16]. Several studies have demonstrated that the microbiome varies between individuals within a species depending on the host’s sex, life stage, or geographic location [17, 18]. Distinct habitats found across geographic space suggest that the environment might shape the mosquito microbiome, and that heterogeneity in the environment may result in the variation seen among mosquito populations [19], potentially contributing to spatial variation in mosquito-borne disease transmission.

**Fig 1:**
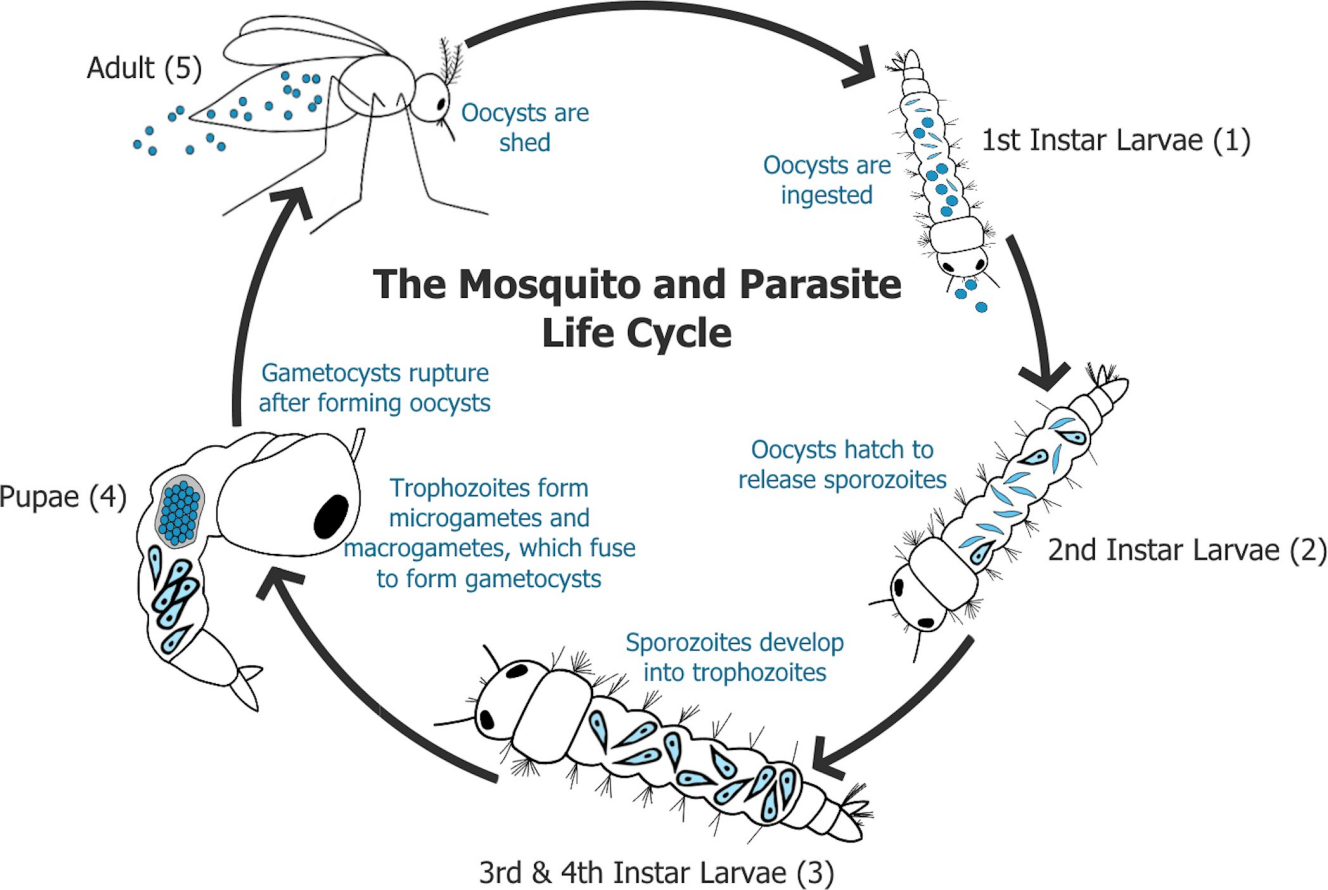
*Ascogregarina taiwanensis* lifecycle. (1) Development of *A*. *taiwanensis* is initiated when oocysts are ingested from the aquatic environment by a 1^st^ instar larvae. (2) Oocysts release sporozoites into host epithelial cells. (3) Sporozoites develop into trophozoites. (4) Before pupation, trophozoites travel to the Malpighian tubules and develop into either micro- and macrogametes that may fuse to form gametocysts. Oocysts develop within the gametocyst. (5) Oocysts are eventually shed into aquatic environment during the transition from pupae to adult. Life-cycle adapted from Tseng, 2007 [28].

Interactions between co-symbiotic microbes within a host might also shape the mosquito microbiome. For instance, recent studies have found that co-occurring bacteria were negatively correlated with other bacterial taxa in medically important mosquitoes [15]. Specifically, *Wolbachia* sp. was found to be negatively correlated with bacterial taxa *Serratia* sp. and *Aeromonas* sp., highlighting the complex interaction networks that may occur among symbionts in the mosquito host [15]. Several *Aedes* sp. mosquitoes are host to an apicomplexan pathosymbiont, *Ascogregarina taiwanensis* [20]. These parasites have a complex life cycle in their mosquito host that begins with the ingestion of oocysts that mature and release sporozoites. These sporozoites invade midgut epithelial cells and develop into trophozoites that migrate selectively to the Malpighian tubules. Here, they develop into macro- and microgametes that fuse to form diploid gametocysts, within which infectious oocysts mature (Fig 1) [21]. Oocysts are eventually shed into the aquatic environment during the metamorphosis transition (pupae to adult), and are ingested by subsequent 1^st^ instar larvae that develop in the same aquatic environment. *A*. *taiwanensis* broadly infect mosquitoes (Diptera: Culicidae) and sand flies (Diptera: Psychodidae), and they are globally distributed [21–24]. The host specificity of *A*. *taiwanensis* remains unresolved. Some studies suggest a high host specificity [25], while others demonstrate contradictory results [26]. Recent studies have found various dispersal strategies used by *A*. *taiwanensis* based on host sex and environmental cues [27]. While Ascogregarine parasites in mosquitoes have served as a model system to investigate the evolutionary biology and physiological impacts of parasitism, their association with the composition of the mosquito microbiome and potential use as a biocontrol [22] remains poorly resolved.

In this study, we investigated specific environmental and ecological factors that influence diversity in the bacteriome of the medically important *Aedes albopictus* mosquito. *Aedes albopictus* is a major vector of rapidly emerging chikungunya virus [29] and is widespread throughout Hawai‘i [30]. Mosquito samples were collected for an entire year across eight sites on Maui, HI. Maui was chosen because it is a high Pacific island (3,055 meters) that has drastic environmental gradients [31] in monthly average temperature (~8°C to 32°C) and rainfall (~0.25mm to 710mm) across relatively short geographical distances (5–60 km). As a result, high oceanic islands such as Maui can serve as a natural model to assess how ecological and environmental factors influence the assembly and composition of the mosquito microbiome. Our results suggest that the bacteriome of *Ae*. *albopictus* on Maui varies greatly among sites but is independent of the gradients in monthly average temperature and rainfall across these sites. In addition, we show that the composition of the *Ae*. *albopictus* microbiome is correlated with the host’s intensity of *A*. *taiwanensis* infection. The resulting data imply that cross-domain interactions in co-symbiosis may be a powerful modulator of the microbiome of a globally-important disease vector.

## Methods

### Mosquito collection

Adult female (n = 96) and male (n = 22) *Ae*. *albopictus* mosquitoes were collected at eight sites across Maui, HI at two-month intervals over the course of a single year during 2017 (S1 and S2 Tables). While collection occurred six times over the course of a year, only five of the collection periods resulted in mosquitoes that were suitable for analysis. Mosquitoes were collected at each site using BG II Sentinel Traps (Biogents, Rogensburg, Germany), powered by Power-sonic batteries (San Diego, CA) and baited with CO_2_ (Airgas, Hawai‘i) and BG lures (BioQuip, Rancho Dominguez, California).

A single BG II Sentinel trap, which tends to capture a majority of host-seeking females [32], was deployed for 24 hours at each site. After the 24 hour collection period, all mosquitoes were removed from the trap and identified by species, sex, and gravid status using an Olympus SZ51 Microscope (Olympus, Tokyo, Japan) and the Darsie and Ward taxonomic key [33]. Gravid status was assessed based on visualizing the size of the abdomen [34]. The number of collected *Ae*. *albopictus* varied across sites and sampling periods (S2 Table). Of the mosquitoes collected, only adults that were alive and intact at the time of trap collection were used to characterize the microbiome. Blood-fed and gravid (i.e., enlarged abdomen) females were excluded from analysis. When possible, 1–10 individual mosquitoes (mean = 2.95, median = 2) were selected per site, sampling period, and collection period and processed individually as samples for analysis (S2 Table). Once identified and selected for analysis, mosquito samples were stored in 70% ethanol and cooled on dry ice until they were returned to the laboratory and stored at -80°C.

Average temperature [35] and rainfall [36] measurements were acquired from each site and collection period from the Climate Atlas of Hawaii (S1 Table). These sites ranged in altitude from 18 to 975 m, and spanned monthly rainfall and temperature gradients of 2.29 to 271.4 mm and 15 to 26°C respectively (S1 Table).

### Sample extraction and sequencing

Before extraction, samples were surface-sterilized by a protocol adapted from previous studies: one 70% ethanol wash, followed by two rinses in sterile 1X PBS [37, 38]. DNA was then extracted and purified using a QIAamp DNA Mini Kit (Qiagen, Hilden, Germany) following the manufacturer’s protocol. Library preparation was done using a modified version of the Earth Microbiome Project 16S V4 protocol [39]. PCR amplification targeted the V4 region of bacterial 16S rRNA by using Earth Microbiome dual indexed barcoded primers. These primers included standard 515F (5’-GTGCCAGCMGCCGCGGTAA-3’) and 806R (5’-GGACTACHVGGGTATCTAAT-3’) 16S primers, along with unique attached barcodes [40] (S5 Table). Each PCR reaction contained the following components: 16.25 μL of nuclease-free water, 5.0 μL of 5X KAPA HiFi Fidelity Buffer (Roche, Basel, Switzerland),1.0 μL of template DNA (approximately 10–87 ng), 0.75 μL of 10 mM KAPA dNTP Mix, 0.75 μL of 10 μM forward primer, 0.75 μL of 10 μM reverse primer, and 0.5 μL of 1 U/μL KAPA HiFI HotStart DNA Polymerase. A no-template PCR reaction was also performed as a negative reagent blank. PCR amplifications were performed in an Applied Biosystems SimpliAmp Thermal Cycler (Thermo Fisher Scientific, Waltham, MA) using the following conditions: initial denaturation at 95°C for 3 min; 35 cycles of denaturation at 98°C for 20 s, annealing at 60°C for 15 s, extension at 72°C for 30 s; and a final extension at 72°C for 30 s. The PCR products were visualized on a 2% agarose gel before being purified and normalized to approximately 1.25 to 2.50 ng/μL using a Just-a-Plate kit (Charm Biotech, Cape Girardeau, MO). Purity and concentration of a subset of samples were assessed with a NanoDrop (Thermo Fisher Scientific, Waltham, MA). A volume of 6 μL from each sample was pooled and then purified using a 1.2X volume of Serapure Beads [41]. The library was checked for quality and quantity using a Bioanalyzer High Sensitivity chip (Agilent Technologies, Santa Clara, CA) run by Advanced Studies in Genomics, Proteomics and Bioinformatics at the University of Hawai‘i at Mānoa. Once quality was confirmed, the library was sequenced (UC Davis Genome Center) using Illumina MiSeq PE300 with a MiSeq reagent kit v3 (Illumina, San Diego, CA).

### Bioinformatics analysis

Raw paired fastq reads were preprocessed using the dada2 R package [42]. Reads were filtered with the filterAndTrim() function. Reads were truncated at position 220 (190 for the reverse read) and discarded if they contained at least one base below quality 2 or a number of expected errors above 3. Denoising was performed using the learnError() and dada() functions with default parameters. Using the mergePairs() function, reads were merged if they overlapped by at least 20 bases, and a maximum of one mismatch was allowed. Mothur was used along with the Silva database (version 132) (downloaded from https://mothur.org/wiki/Silva_reference_files) to align and annotate the sequences. Sequences with a start or stop position outside the 5^th^ - 95^th^ percentile range (over all sequences) were discarded. Potential chimeras were removed with chimera.uchime() and clustered at 99% similarity thresholds with chimera.vsearch(). Taxonomies were assigned using classify.seqs() and classify.otus(). The lulu R package was used to refine operating taxonomic units (OTUs) [43]. Two OTUs were merged if all the three following conditions were satisfied: *i*) they co-occur in every sample, *ii*) one of the two OTUs has a lower relative abundance than the other in every sample, and *iii*) they share a sequence similarity of at least 99%. Finally, all singletons (OTUs with reads in only one sample) and OTUs with no annotation at the kingdom level were removed.

After quality control, sequencing resulted in 19.5 million reads (per sample mean: 79,864; per sample median: 76,085). OTUs that represented less than 0.01% of reads and had less than 5% prevalence were excluded from further analysis. Samples with less than 4,000 reads were also removed. OTUs found in the reagent negative-controls were also removed from analysis as they likely represent contaminants along the molecular analysis pipeline. These included the taxa *Delftia* sp., *Herbaspirillum* sp., and Sandaracinaceae sp. Overall, 18 OTUs were used for downstream analysis, including two *Wolbachia* sp. strains (wAlbA and wAlbB), one *Asaia* sp., four bacteria from Enterobacteriaceae, and nine other taxa in the bacterial families *Burkholderiaceae*, *Xanthobacteraceae*, *Pseudomonadaceae*, *Streptomycetaceae*, *Staphylococcaceae*, *Xanthomonadaceae*, *Beijerinckiaceae*, *Rhizobiaceae*, and *Sphingomonadaceae*.

### Estimation of *Ascogregorina taiwanensis* load

Total *Ascogregorina taiwanensis* infection intensity was assessed by qPCR using custom-designed primers (AT_SHORT_2_F: 5’-TCGATGAAGGACGCAGCTTA-3’, AT_SHORT_2_R: 5’-AGGCACTGAACTGGACATACT-3’) based off of an *A*. *taiwanensis* reference sequence (GenBank AY326461.1). Each qPCR reaction contained the following components: 5.0 μL of PowerUp SYBR Green Master Mix (Applied Biosystems, Foster City, CA), 3.0 μL of nuclease-free water, 1.0 μL of template DNA (approximately 10–87 ng), 0.5 μL of 10 μM forward primer, and 0.5 μL of 10 μM reverse primer. The reactions were performed using an Applied Biosystems StepOnePlus Real-Time PCR System (Thermo Fisher Scientific, Waltham, MA) using the following conditions: UDG activation at 50°C for 2 min; Dual-Lock DNA polymerase at 95°C for 2 min; and 40 cycles of denaturation at 95°C for 15 s, annealing at 56°C for 15s, and extension at 72°C for 1 min. Fluorescence readings were taken at the 56°C annealing step for each cycle. A melt curve was performed according to PowerUp SYBR protocol to confirm the specificity of amplification. The Cycle threshold (Ct) values were used to measure *A*. *taiwanensis* infection intensity and were obtained assuming a delta Rn fluorescence threshold of 0.25. Samples were considered negative if the cycle threshold (Ct) value was greater than 38, or if the melt curve did not align with the positive control.

### Estimation of *Wolbachia* load

Total *Wolbachia* sp. load was quantified through qPCR using *Wolbachia*-specific primers (*W*-Spec-16S-F: 5’-CATACCTATTCGAAGGGATA-3’ and W-Spec-16S-R: 5’-AGCTTCGAGTGAAACCAATTC-3’) (Werren and Windsor, 2000). Primers of the actin gene, alb-act-F (CCCA CACAGTCCCCATCTAC) and alb-act-F (CGAGTAGCCACGTTCAGTCA) (Calvitti et al., 2015), were used to quantify host genomic copies. Each qPCR reaction contained the following components: 5.0 μL of 2X PowerUp SYBR Green Master Mix, 1.0 μL of nuclease-free water, 1.0 μL of 10 μM forward primer, 1.0 μL of 10 μM reverse primer, and 2.0 μL of template DNA (approximately 10–87 ng). The reactions were performed using an Applied Biosystems StepOne Plus Real-Time PCR System (Thermo Fisher Scientific, Waltham, MA) using the following conditions: UDG activation at 50°C for 2 min; Dual-Lock DNA polymerase at 95°C for 2 min; and 40 cycles of denaturation at 95°C for 15 s and annealing/extension at 60°C for 1 min. Fluorescence readings were taken at the 60°C annealing/extension step for each cycle. A melt curve stage was performed according to PowerUp SYBR protocol to confirm the specificity of amplification. The Ct values were used to measure load intensity and were obtained assuming a delta Rn fluorescence threshold of 0.3. The relative abundance (i.e. *Wolbachia* index) was estimated as a ratio of the inverse Wolbachia Ct value to the inverse Ct of the single copy mosquito gene, actin. qPCR reactions with no detected Wolbachia template received a Ct value of 41 (total cycles in the reaction plus one).

### Estimation of *Asaia* load

Total *Asaia* sp. load was quantified using custom-designed primers (ASAS_1_F: 5’-CGGCAACCTGGCTCATTAC-3’, ASAS-1-R: 5’-ACATCCAGCACACATCGTTTAC-3’) based off a partial 16S rRNA sequence obtained from lab cultures isolated from *A*.*albopictus* midguts. Each qPCR reaction contained the following components: 5.0 μL of 2X PowerUp SYBR Green Master Mix, 3.0 μL of nuclease-free water, 0.5 μL of 10 μM forward primer, 0.5 μL of 10 μM reverse primer, and 1.0 μL of template DNA (approximately 10–87 ng). The reactions were performed using an Applied Biosystems StepOne Plus Real-Time PCR System Thermo Fisher Scientific, Waltham, MA) using the following conditions: UDG activation at 50°C for 2 min, Dual-Lock DNA polymerase at 95°C for 2 min; and 40 cycles of denaturation at 95°C for 15 s, annealing at 58°C for 15 s, and extension at 72°C for 1 min. Fluorescence readings were taken at the 58°C annealing step for each cycle. A melt curve stage was performed according to PowerUp SYBR protocol to confirm the specificity of amplification. The relative abundance (i.e. *Asaia* index) was estimated as a ratio of the inverse *Asaia* Ct value to the inverse Ct of the single copy mosquito gene, actin. qPCR reactions with no detected *Asaia* template received a Ct value of 41 (total cycles in the reaction plus one).

### Statistical analysis

Generalized linear mixed models implemented in the package glmmTMB in program R were used to test a relationship between species richness (the number of bacterial OTUs per mosquito) and the fixed effects of rainfall, temperature, and *A*. *taiwanensis* infection. Linear mixed models implemented in package lme4 [44] in program R were used to test a relationship between logit transformed species evenness (the Simpson’s index of each mosquito’s microbiota, estimated with the function “diversity” in the package vegan) and the fixed effects of rainfall, temperature, and *A*. *taiwanensis* infection [45]. Site and month were included as random effects in these models. Richness was measured as the total number of OTUs in a sample, and unbalanced read counts across samples were controlled for with the “offset” function in the base package of R. The significance of individual independent effects on α-diversity indices was assessed through a log-likelihood ratio test that compared a full model with a nested model that lacked an independent variable of interest. We assumed the log-likelihood approximates a Chi-square distribution.

We used zero-inflated generalized linear mixed models implemented in the package *glmmTMB* [46] in program R to test for differences in the composition of the microbiota from host mosquitoes across sites and months in relation to rainfall, temperature, mosquito sex, and intensity of *A*. *taiwanensis* infection. The relative abundance of each bacterial taxon in each mosquito individual was calculated to create a single response variable. To accomplish this, the out community matrix was transformed from wide form (in which each row dictates an individual host mosquito coupled with columns populated by read counts of specific symbionts in that host) to long form (in which each row is a unique combination of an individual host and a bacterial taxon coupled with columns that show the read count of the bacteria in that host individual). As microbiome data is inherently composite in nature [47], and each read count for a given taxon should only be interpreted in the context of the total reads for that sample, the models were fitted assuming a binomial error distribution. Given that several observations (rows) in this dataset are tied to a specific symbiont taxon (in this data, there are 118 observations per individual bacterial taxon), symbiont taxa (18 levels in this dataset) was modeled as a random intercept in all candidate models. In addition, as the proportional data (reads from a specific bacterium of the total bacteria reads) were highly over-dispersed, an observation-level random intercept was fit to allow for overdispersion in all candidate models.

We use random slopes and random interactions with their corresponding fixed and main effects to allow the relative abundance of each OTU to independently change in response to explanatory variables in the model. The effect of monthly average rainfall, monthly average temperature, and *A*. *taiwanensis* infection intensity status on the microbiota composition were tested by modeling random slopes for each symbiont taxon, while site, month, and sex were tested as random interactions. All random slopes were modeled as uncorrelated with the random intercept. Zero-inflation was allowed to vary across the levels of the site × OTU taxa random interaction, and the random intercept within symbiont taxa in each model. The significance of these random slopes and interactions was assessed to test for changes in the composition of the microbiome using log-likelihood ratio tests. The conditional modes of these responses reveal effect sizes. In addition, random interactions of “site x Ascogregarina presence”, “OTU x Ascogregarina presence x sex”, and “OTU x Ascogregarina presence x site” were also tested to assess whether the effects of Ascogregarine infection were consistent across levels of “sex” and “site” (S1 Text and S7 Table).

In addition to the analyses above, we describe the data with the average proportion of reads for each OTU, which was calculated for individual mosquitoes at each sampling site. The OTU relative abundances of each symbiont were converted into presence-absence data for each site, and the percentage of mosquitoes hosting each symbiont at each site were calculated. The presence-absence data for *A*. *taiwanensis* infection were used to calculate the prevalence of individual mosquitoes at each site that were positive for the non-metazoan parasite (S5 Table). We use the best-fit model to produce predicted probabilities for each symbiont taxa in a mosquito host with an intense Ascogregarine infection (i.e. the lowest Ct value in these data) and a host with no detectable Ascogregarine infection (i.e. no detected amplification through qPCR). We then use these predicted probabilities to estimate odds ratios to demonstrate the direction and effect size for each symbiont taxa. Mixed models implemented in the package *glmmTMB* in program R were also used to interrogate the probability of *A*. *taiwanensis* infection in relation to the fixed effects of rainfall, temperature, *Wolbachia* sp. index, *Asaia* sp. index, and random effects of site, month, and sex. Effects were tested with a log-likelihood ratio test, as previously described.

## Results

### The *Aedes albopictus* bacteriome

**Fig 2:**
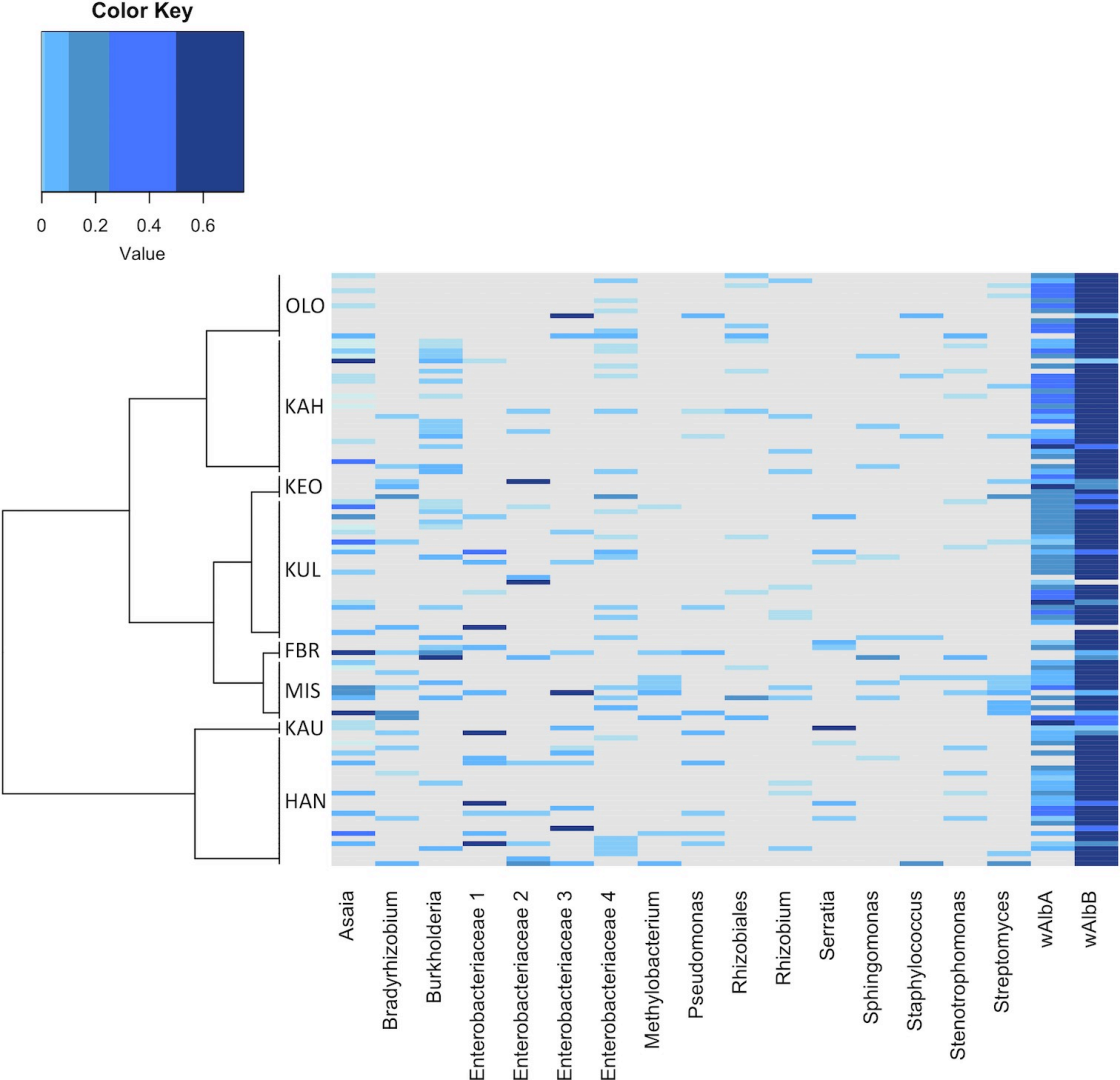
Heatmap showing the prevalence of bacterial taxa within an individual mosquito distributed across sites. Cell values are calculated as proportions across rows. The site dendrogram was estimated with Euclidean distances based on geo-spatial data. Sites abbreviations: Forest Bird Recovery (FBR), Hana (HAN), Kahului (KAH), Kaupo (KAU), Keokea (KEO), Kula (KUL), Maui Invasive Species Council (MIS), Olowalu (OLO) (S1 Table). Enterobacteriaceae taxonomic names had multiple hits when blasted against the NCBI GenBank database and have been given family names for clarity. Enterobacteriaceae 1–4 were most similar to sequences identified as *Klebsiella* sp. and *Enterobacter* sp.; *Pantoea* sp.; *Cedecea* sp. or *Klebsiella* sp.; and *Yersinia* or *Serratia*, respectively.

A total of 716 bacterial OTUs were identified from 118 adult *Ae*. *albopictus* mosquitoes across the island of Maui (S1 Table), 18 of which had a prevalence greater than 0.05% (Fig 2; S3 and S4 Tables). Among the 18 OTUs, three bacterial Phyla (Proteobacteria, Acitinobacteria, and Firmicutes) and twelve bacterial families (*Anaplasmataceae*, *Acetobacteraceae*, *Enterobacteriaceae*, *Burkholderiaceae*, *Xanthobacteraceae*, *Pseudomonadaceae*, *Streptomycetaceae*, *Staphylococcaceae*, *Xanthomonadaceae*, *Beijerinckiaceae*, *Rhizobiaceae*, and *Sphingomonadaceae*) were detected (Fig 2). Within the bacterial family *Anaplasmataceae*, two *Wolbachia* sp. lineages, *Wolbachia* sp. wAlbB, and *Wolbachia* sp. wAlbA, accounted for 89% of the sequencing reads, and were found in 99% of samples. The symbiont within the genus *Asaia* of the Acetobacteriaciae family accounted for 3.9% of overall sequencing reads and was found in 30% of samples. Several other OTUs belonged to the family Enterobacteriaceae. With the exception of a *Serratia marcescens* (7% of samples, 1.7% sequencing reads), the genus of these Enterobacteriaceae taxa could not be determined conclusively. These taxa were labelled Enterobacteriaceae 1–4. The most supported taxonomic identity (as indicated by the Basic Local Alignment Search Tool on GenBank) is as follows: Enterobacteriaceae 1 (likely *Klebsiella* sp. or *Enterobacter* sp., 13% of samples, 1.5% sequencing reads), Enterobacteriaceae 2 (likely *Pantoea* sp., 10% of samples, 1.5% sequencing reads), Enterobacteriaceae 3 (likely *Cedecea* sp. or *Klebsiella* sp., 11% of samples, 0.97% sequencing reads), and Enterobacteriaceae 4 (likely *Yersinia* sp. or *Serratia* sp., 22% of samples, 0.05% sequencing reads). The bacterial genera *Burkholderia* sp. was detected in 25% of samples and 0.26% of sequencing reads. Other OTUs were detected in the samples at varying levels, such as a Rhizobiales (9% of samples, 0.07% sequencing reads), *Bradyrhizobium* (14% of samples, 0.05% sequencing reads), *Pseudomonas* sp. (9% of samples, 0.05% sequencing reads), *Streptomyces* sp. (13% of samples, 0.04% sequencing reads), *Staphylococcus* sp. (5% of samples, 0.03% sequencing reads), *Strenotrophomonas* sp. (11% of samples, 0.02% sequencing reads), *Methylobaterium* sp. (7% of samples, 0.02% sequencing reads), Rhizobium (10% of samples, 0.008% sequencing reads), and *Spingomonas* sp. (7% of samples, 0.01% sequencing reads). The OTUs Rhizobiales and Rhizobium could not be classified to lower taxonomic rankings.

### Effects on α-diversity of the *Aedes albopictus* microbiome

When the *Ae*. *albopictus* microbiome was defined as a set of the 18 most prevalent bacterial taxa, the average (± standard error of the mean) species richness was 3.9 ± 0.14 and evenness (Simpson’s index) was 0.30 ± 0.016. Temperature (p-value = 0.37), rainfall (p-value = 0.15), and *A*. *taiwanensis* infection (p-value = 0.09) were not significantly associated with the richness of mosquito microbiotas (generalized negative binomial mixed model, Table 1). However, richness varied significantly across geography (site: p-value = 0.016), and marginally insignificantly with time (month: p-value = 0.074).

**Table 1:** The estimates and standard errors for variables included in the full linear mixed model to explain variation in species richness*.

Fixed Effects	Estimate	Standard error
Intercept	-8.4	1.52
Temperature	-0.059	0.064
Rainfall	0.0056	0.037
*A*. *taiwanensis*	0.022	0.013
Conditional Model		
Random Effects	Variance	Standard Deviation
Site	0.17	0.42
Month	0.08	0.29

* The species richness was used as response variables in the linear mixed model.

Temperature (p-value = 0.45), rainfall (p-value = 0.47), and *A*. *taiwanensis* infection level (p-value = 0.66) were not associated with rarefied evenness (Table 2). The random effects of site (p-value = 1) and month (p-value = 0.82) were also not associated with rarefied evenness, offering no support that rarefied evenness varied with any variable included in the analysis.

**Table 2:** The estimates and standard errors for variables included in the full linear mixed model to explain variation in species evenness.

Full Model	Estimate	Standard Error
Intercept	-0.29	1.5
Temperature	-0.048	0.063
Rainfall	-0.0018	0.0038
*A*. *taiwanensis*	0.013	0.019
Conditional Model		
Random Effects	Variance	Standard Deviation
Site	0.12	0.35
Month	0	0

### Modeling β-diversity of the *Ae*. *albopictus* microbiome

**Fig 3:**
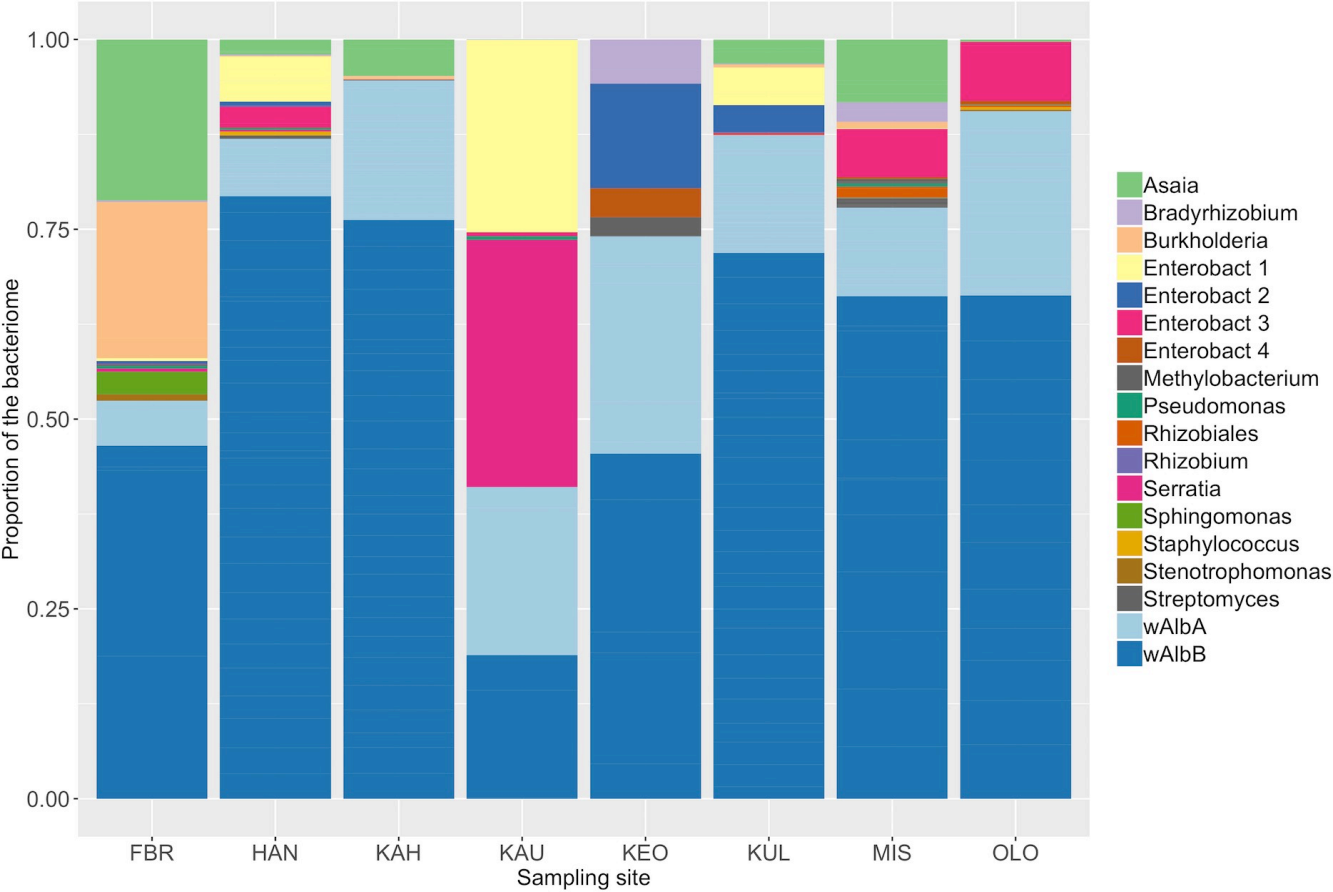
The composition of the bacteriome of *Ae*. *albopictus* among different sampling sites on Maui, HI. This figure represents the bacteriome of mosquitoes collected over entire study (n = 118): FBR (n = 4), HAN (n = 26), KAH (n = 27), KAU (n = 3), KEO (n = 5), KUL (n = 28), MIS (n = 12), OLO (n = 13).

The first stage of this modelling effort tested spatiotemporal variation in the composition of the microbiome, while controlling for variables that vary at the level of individual mosquito hosts (i.e. Ascogregarine infection and host sex). The *Ae*. *albopictus* microbiome varied across sites (Fig 3 and Table 3; random interaction between site and OTU, p-value = 0.0015). However, the effect of month was not significantly associated with variation in the composition of the mosquito microbiome (p-value = 0.81). *Wolbachia* species wAlbB and wAlbA were the symbionts with the greatest relative abundance (89% of reads). *Wolbachia* sp. were found at all sites across Maui, with their presence in mosquitoes varying from 50–100% across sampled sites (S4 Table). *Asaia* sp. was the next most prevalent species, found in all locations except Keokea with an average reads percentage of 0.021–21% (S3 Table) and prevalence of 0–66% across sites (S4 Table). *Serratia* sp. prevalence varied from 0–33%, and was found in 50% of the sites sampled, with Kaupo containing the highest proportion of reads (S3 and S4 Tables). Other environmentally acquired symbionts like Enterobacteriaceae 1–3 were detected across 0–33% of sites (S4 Table). The highest proportion of Enterobacteriaceae 1 reads was detected in Kaupo (25%), while Keokea and Olowalu contained the highest proportions of Enterobacteriaceae 2 (14%) and Enterobacteriaceae 3 (7.8%), respectively (S3 and S4 Tables). The prevalence of the other symbionts varied across sites from 0 to 50% (S3 and S4 Tables).

**Table 3:** The estimates and associated standard errors of fixed effects, and the variance and associated standard deviation of the random effects in a mixed effects model that only included significant effects in the analysis, which interrogates the change in the composition of the microbiome within *Ae*. *albopictus* mosquitoes. *.

Full Model	Estimate	Standard error
Intercept	-4.7	0.50
*A*. *taiwanensis*	0.13	0.20
Conditional Model		
Random Effects	Variance	Standard Deviation
Site	0.16	0.40
OTU	3.5	1.9
Site x OTU	0.97	0.99
*A*. *taiwanensis*	0.35	0.59

* Best fit model included a row ID variable row ID to control for overdispersion and fixed effect for *A*. *taiwanensis* infection intensity.

In the second stage of this modeling effort, environmental variables (temperature and rainfall) were tested as potential drivers of the significant spatial variation seen in the microbiome, while controlling for the same variables that vary at the level of individual hosts. Although rainfall and temperature varied across sites and over sampling periods (S1 Table), these environmental variables were not associated with microbiome composition in these data (rainfall p-value = 1.0, temperature p-value = 0.94).

**Fig 4:**
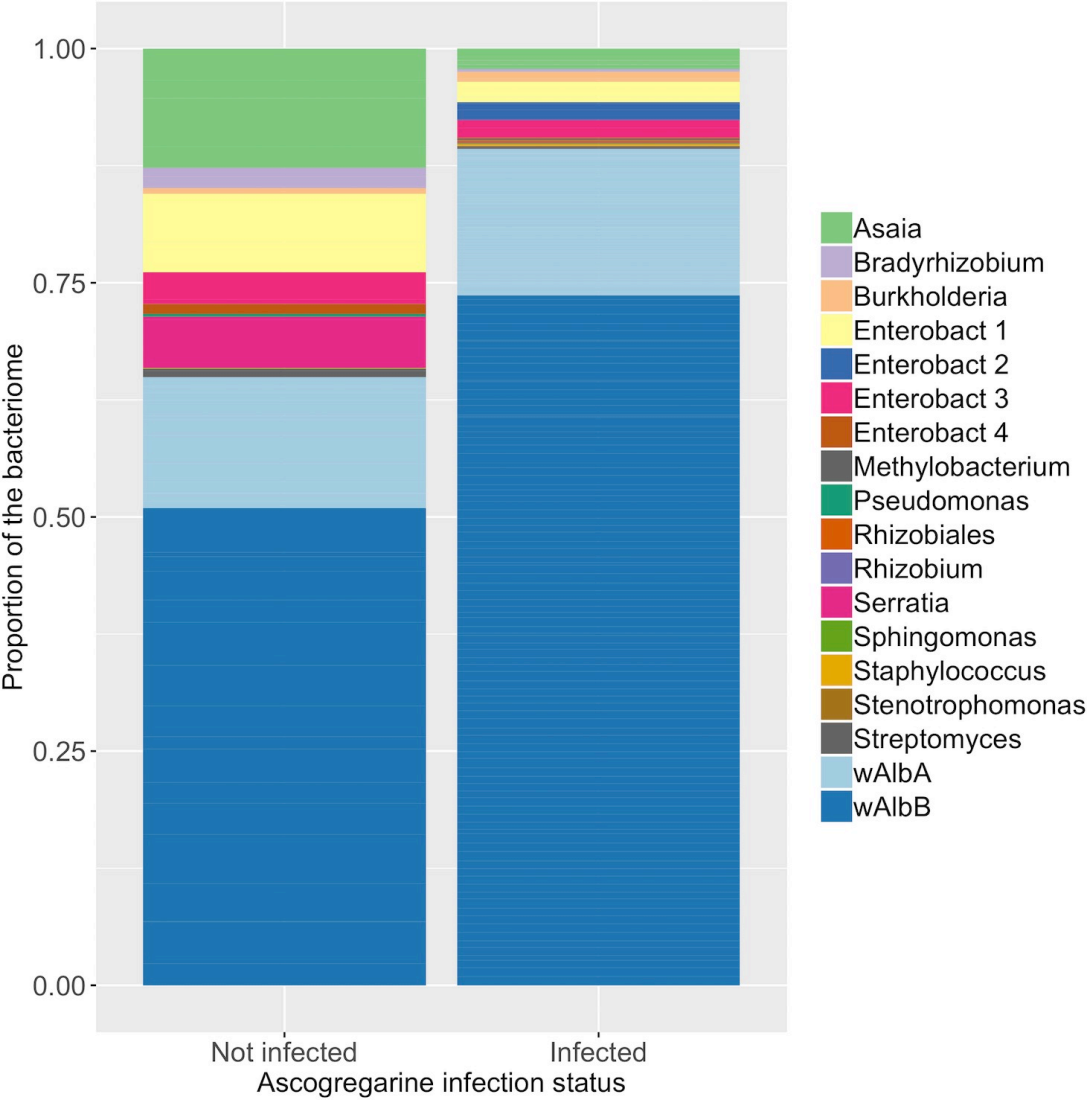
The composition of the *Ae*. *albopictus* bacteriome between *A*. *taiwanensis*-infected (n = 99) and uninfected (n = 19) mosquito individuals.

Lastly, the individual level effects of Ascogregarine infection and host sex were tested as potential drivers of microbiome variation, while controlling for spatial variation. The composition of the microbiome was significantly associated with *A*. *taiwanensis* infection levels (Table 3; p-value = 0.01). The probability of sampling wAlbB increased by a factor of 3.2 under intense *A*. *taiwanensis* infection relative to hosts with no detectable infection, while the probability of sampling wAlbA remained relatively unchanged (Table 4). Similarly, the odds ratios indicated that the probability of sampling Enterobacteriaceae 2, dramatically increased 7.5 times in the presence of an intense *A*. *taiwanensis* infection (Table 4). This was not consistent across the family, however, as Enterobacteriaceae 3 (OR = 1.1) had a relatively modest increase while Enterobacteriaceae 1 (OR = 0.06) and *S*. *marsescens* (OR = 0.1) had a decrease in the presence of *A*. *taiwanensis* (Table 4). Other groups, such as *Asaia* (OR = 0.41) and Bradyrhizobium (OR = 0.26) also had a lower probability of being detected in mosquitoes with *A*. *taiwanensis* infection (Fig 4, Table 4). The odds ratios of other symbionts ranged between 0.05–1.5, suggesting that they did not change substantially in the host’s microbiome across Ascogregarine-infection intensity (Fig 4, Table 4). Finally, there was no relationship between sex of the mosquito and the microbiome composition (p-value = 0.37).

**Table 4:** Odds ratios indicating predicted probabilities of sampling bacterial taxa when mosquitoes are highly infected with *A*. *taiwanensis* or not infected.

	Predicted probability from best fit model		
	High intensity Infection	No detectable Infection	Odds ratio
wAlbB	0.71	0.44	3.2
wAlbA	0.13	0.12	1.1
Asaia	3.3x10^-3^	8.0x10^-3^	0.41
Serratia	8.1x10^-4^	8.7x10^-3^	0.1
Enterobacteriaceae 1	2.7x10^-3^	4.1x10^-2^	0.06
Enterobacteriaceae 2	8.8x10^-3^	1.2x10^-3^	7.5
Enterobacteriaceae 3	6.7x10^-3^	6.0x10^-3^	1.1
Burkholderia	5.1x10^-3^	5.5x10^-3^	0.93
Enterobacteriaceae 4	8.5x10^-4^	3.4x10^-3^	0.25
Rhizobiales	6.9x10^-4^	8.6x10^-4^	0.80
Bradyrhizobium	1.7x10-3	6.6x10^-3^	0.26
Pseudomonas	1.0x10^-3^	1.6x10^-3^	0.65
Streptomyces	1.3x10^-3^	4.0x10^-3^	0.32
Staphylococcus	9.5x10^-4^	8.8x10^-4^	1.1
Stenotrophomonas	5.4x10^-4^	4.3x10^-4^	1.3
Methylobacterium	1.4x10^-3^	5.4x10^-4^	2.6
Rhizobium	3.1x10^-4^	5.6x10^-4^	0.55
Sphingomonas	5.1x10^-4^	6.1x10^-4^	0.84

### Drivers of *Ascogregarina* infection

Shifts in the relative abundance of a focal symbiont (e.g. wAlbB) may be due to a change in the absolute abundance of that taxon, or the absolute abundance of the other taxa in the microbiome. To clarify the observation that the wAlbB and several environmentally acquired bacteria (e.g. *Asaia* sp.) change in relative abundance with the mosquito microbiome with *A*. *taiwanensis* infections status, we tested whether *A*. *taiwanensis* infection is correlated with an index of absolute abundance of *Wolbachia* sp. and *Asaia* sp. bacteria, while controlling for other environmental factors that might influence infection. Temperature (p-value = 0.96), rainfall (p-value = 0.65), and the index of *Wolbachia* sp. absolute abundance (p-value = 0.60) were not significantly associated with the probability of *A*. *taiwanensis* infection (Table 5). However, there was a marginally significant association for the index of *Asaia* sp. absolute abundance (p-value = 0.077), suggesting low *Asaia* sp. abundance in Ascogregarine-infected mosquitoes. For example, mosquitoes with an *Asaia* sp. index of zero (i.e. no detectable *Asaia* infection) were 8.3X more likely to harbor an *A*. *taiwanensis* infection than mosquitoes that had an *Asaia* index of 1. The range of the *Asaia* sp. index in these data was 0.50–1.51, so an increase of 1 roughly corresponds to the change in *Asaia* sp. abundance from the least to the greatest values in the sample. The random intercept of site (p-value = 1), month (p-value = 0.50), and sex (p-value = 1) were not associated with *A*. *taiwanensis* presence, suggesting that the prevalence of *A*. *taiwanensis* is similar among collection sites, across months, and between the sexes (Table 5).

**Table 5:** The estimates and standard errors of fixed effects and the variances and standard deviation of the random effects in a mixed effect model that interrogates the change in *A*. *taiwanensis* infection between mosquitoes.

Full Model	Estimate	Standard error
Intercept	2.9	3.4
Temperature	0.0059	0.14
Rainfall	-0.0036	0.0077
*Wolbachia* sp. Index	0.73	1.4
*Asaia* sp. Index	-2.6	1.5
Conditional Model		
Random Effects	Variance	Standard Deviation
Site	0.43	0.65
Month	1.01 x 10−^9^	3.2 x 10^−5^

## Discussion

The microbiome of a mosquito alters the physiological traits of vectors that can scale to impact its vectorial capacity [19, 48]. While many studies have shown that the microbiome varies within and between mosquito species [49], the specific factors that contribute to this variation are poorly resolved. The aim of this study was to understand how certain biotic and abiotic factors influence the composition of the microbiome of *Ae*. *Albopictus*, a globally important vector of chikungunya virus [50, 51]. Broadly, the data suggest that the composition of *Ae*. *Albopictus* bacteriomes on Maui are associated with a eukaryotic and parasitic co-symbiont, *Ascogregarina taiwanensis*. These microbiotas also change in composition across sites, but these changes are independent of stark environmental gradients of temperature and precipitation (S1 Table, S8 Table).

Previous studies have described interactions between co-symbionts within a mosquito host in structuring the overall host’s microbiota [52–54]. For instance, the bacterial genera *Asaia* sp. may act antagonistically to impede the vertical transmission of *Wolbachia* sp. in *Anopholes* sp. mosquitoes [15], and is generally negatively correlated with the presence of *Wolbachia* sp. endosymbionts. Other studies extend these relationships to interdomain interactions. For instance, *Serratia marcescens* strains and *Chromobacteriaum Csp_P* impact the development of *Plasmodium* sp. in *Anopholes* sp. mosquitoes [55]. Our data suggest that the level of infection with another ampicomplexan parasite, *A*. *taiwanensis*, is significantly associated with the bacteriome of *Ae*. *albopictus*. Mosquitoes with *A*. *taiwanensis* infection were more likely to contain several bacterial symbionts including wAlbB and Enterobacteriaceae bacteria that are most similar to *Pantoea* sp. and *Burkholderia* sp. Mosquitoes without an *A*. *taiwanensis* infection, were more likely to contain other symbionts, including *Asaia* sp., *Serratia marcescans*, and another Enterobactericecae bacteria that was most similar to *Klebsiella* sp. The results indicate that while a dramatic increase in relative abundance of wAlbB in *A*. *taiwanensis-*infected mosquitoes was observed, the total abundance of wAlbA and wAlbB (i.e., *Wolbachi*a index) remained unchanged. This might be associated with shifts in the relative abundance of wAlbA relative to wAlbB between *A*. *taiwanensis-*infected and uninfected mosquitoes. Alternately, the apparent increase in relative abundance may also be due to a reduction in the abundance of environmentally acquired symbionts that compete for reads with *Wolbachia* sp. Indeed, mosquitoes that lacked *A*. *taiwanensis* infections had an increase in the index of absolute *Asaia* sp. abundance. Although this effect was marginally insignificant, the trend of greater *Asaia* sp. absolute abundance in mosquitoes without *A*. *taiwanensis* infection comports with a hypothesis that the increase in apparent relative abundance of *Wolbachia* sp. is actually associated with a decrease abundance in some environmentally acquired bacterial symbionts. Further work (see below) is necessary to explore this relationship.

The co-occurrence of *A*. *taiwanensis* life-cycle stages with environmentally acquired symbionts like *Asaia* sp. in specific organs and tissues provides ample opportunity for both direct and indirect interactions between these bacterial and eukaryotic symbionts. The infection of *A*. *taiwanensis* occurs in the early aquatic life stages of mosquitoes [20, 21] and thus proceeds through several tissues in the mosquito that are also important for the persistence and transstadial transmission of environmentally acquired bacterial symbionts, and may explain the compositional changes in the environmentally acquired bacterial microbiome [20, 21, 56]. For instance, the initial site of *A*. *taiwanensis* infection is the mosquito midgut, which harbors a diverse assemblage of symbionts and is a major site of primary colonization of environmentally acquired bacteria in the mosquito [8, 13]. In addition, the Malpighian tubules, which harbor *A*. *taiwanensis*, are conserved during metamorphosis at the pupal stage and have been implicated in the transstadial transmission of symbionts acquired during the larval stage to the adult stage, where disease transmission occurs [57].

Cumulatively, these data highlight the potential for interdomain interactions that may contribute to the diversity of the *Ae*. *albopictus* microbiome. Multiple mechanistic hypotheses might explain the apparent inter- and intra-domain associations identified in this study. Previous studies suggest that *Wolbachia* are associated in numerous interactions with other symbionts [15, 58], either directly through co-exclusion with other bacterial symbionts or indirectly via immune activation pathways. Since *Wolbachia* sp. colonize their host during embryogenesis, these results might imply that *Wolbachia* facilitate *A*. *taiwanensis* infection. However, the similar absolute abundance of *Wolbachia* sp. between Ascogregarine-infected and non-infected mosquito hosts do not support this hypothesis. Conversely, *A*. *taiwanensis* infection may inhibit environmentally acquired symbionts, either directly via mechanical disruption of resident bacterial communities or indirectly via modulation of immune pathways such as the immune deficiency (IMD) and Toll pathways [59]. For example, the response to *A*. *taiwanenesis* infection might potentially induce the release of antimicrobial peptides (AMP) or inhibit C-type lectins (mosGCTLs) that result in the reduction of environmentally acquired symbiont colonization [59]. Direct or indirect mechanisms likely have cascading effects that influence the overall composition of the *Ae*. *albopictus* microbiome and should be addressed in future studies. Ultimately, a controlled fully factorial experiment that manipulates infection with *A*. *taiwanensis*, *Wolbachia* sp., and other environmentally acquired bacterial symbionts would further characterize the direct and indirect interactions between co-occurring taxa, and might resolve the ecological mechanisms by which inter- and intra-domain interactions contribute to the diversity of the microbiome in medically important mosquito vectors.

Several studies have shown that the ecological habitat in which mosquitoes develop may impact the composition of their microbiome [14, 37, 60]. For instance, *Culex nigripalpus* mosquitoes have been shown to display distinct microbiome compositions based on geographic locations [18]. Similarly, our data suggest that bacterial microbiota of *Ae*. *albopictus* varies among sampling locations. However, despite stark environmental gradients in temperature and rainfall that varied spatiotemporally (S1 Table), these environmental variables were uncorrelated with changes in the composition of the *Ae*. *albopictus* bacteriome in this study. We originally hypothesized that distinct habitats and environments would create a distinct source of species pools of symbionts that are available to colonize mosquitoes, and that this would contribute to variation in the mosquito microbiome across space. A lack of correlation between temperature and rainfall, and the composition of the mosquito microbiome is surprising since both these environmental variables typically have strong impacts on mosquitoes directly and structure the broad ecological communities that include local mosquito populations [61–64]. Furthermore, temperature itself strongly influences bacteria replication rates [65]. Other environmental variables such as land use could play a stronger role in the heterogeneity seen in microbiomes. For example, land use practices are expected to affect biogeochemical cycling and microbiome compositions of soil [66]. Similarly, these types of effects might modulate the microbiomes of different mosquito populations in urban, rural, agricultural, and natural environments, where land use and management practices vary. Alternatively, the variation in the mosquito microbiome across space might also be due to ecological drift that results from dispersal limitation and weak niche effects. Additional studies across a larger and finer spatial scale should explore how environmental variables like temperature, elevation, rainfall, land use, and dispersal limitation might contribute to variation in the mosquito microbiome. The elevated islands of Hawai‘i, with their large environmental gradients over short geographic scales and changing topography, remain a particularly valuable natural setting for these types of studies.

This study has identified two important factors that influence the *Ae*. *albopictus* microbiome: geographic location and Ascogregarine infection. We recognize that a limitation of this study was the relatively low number of mosquitoes per geographic location and sampling events across space and time. Ideally, future studies would include higher sampling per geographic location and additional geographic sites to further assess explicit drivers of mosquito microbiome assembly. Clarifying which explicit environmental and ecological characteristics of geographic location that impact the structure and function of the mosquito microbiome is an essential area for future research and will inform our understanding of how vectorial capacity changes across landscapes. This study, and future studies with a similar focus, may shed light on niche effects that drive the assembly of the vector microbiome, perhaps enhancing the ability to predict vector-borne disease outbreaks. In addition, understanding the role of niche effects [67] and dispersal limitation [68] in structuring mosquito microbiotas could inform the development of future paratransgenic strategies that rely on modified bacteria to suppress pathogen carriage or transmission by a vector [69]. Indeed, understanding complex microbial interactions is essential for developing a successful and stable paratransgenic platform [69]. For instance, strong niche effects that modulate bacterial symbiont fitness in its hosts are expected to limit the distribution of the symbionts among mosquito vectors in a complex landscape. Cumulatively, understanding the explicit drivers of the mosquito microbiome assembly may have important implications in lowering the global burden of mosquito-borne disease.

## Supporting information

S1 Text Investigating random interactions to assess overall changes in the composition of the mosquito microbiome. (DOCX)

S1 Table Geographical coordinates, average temperature, and rainfall at eight sites from which adult *Ae*. *albopictus* mosquitoes were collected in Maui, HI during five sampling periods. Average temperature and rainfall measurements were acquired from Climate Atlas of Hawaii. GPS coordinates were gathered at each collection site using a Garmin eTrex touch.
(XLSX)

S2 Table Number of *Ae*. *albopictus* specimens selected for analysis from the pool of mosquitoes collected across sites and sampling periods. Mosquitoes that were subjected to 16s rRNA sequencing at the collection sites and months. Mosquitoes were collected every two months with BG II sentinel traps.
(XLSX)

S3 Table The average proportion of reads for each OTU found in *Ae albopictus* adults sampled from each site in Maui, HI over a one-year period. The average proportion of reads for each OTU, which was calculated for individual mosquitoes at each sampling site.
(XLSX)

S4 Table The percentage of each OTU found in *Ae*. *albopictus* adults sampled from each site on the island of Maui over a one-year period. The OTU raw abundances of each symbiont were converted into presence-absence data for each site and the percentage of mosquitoes hosting each symbiont at each site was calculated.
(XLSX)

S5 Table The percentage of individual *Ae*. *albopictus* adults that were positive for *Ascogregarina taiwanensis* across each site. The presence-absence data for *Ascogregarina taiwanensis* infection was used to calculate the percentage of sites that were positive.
(XLSX)

S6 Table Sequences and barcodes used for sequencing. Library preparation was done using a modified version of the Earth Microbiome Project 16S V4 protocol. These primers included standard 515F and 806R 16S along with unique attached barcodes.
(XLSX)

S7 Table The estimates, standard errors, and p-values of fixed effects and variances, standard deviation, and associated p-values of the random effects in a mixed effects model that interrogates the change in the composition of the microbiome within *Ae*. *albopictus* mosquitoes. Using a mixed effects model, random interactions of “site x Ascogregarina presence”, “OTU x Ascogregarina presence x sex”, and “OTU x Ascogregarina presence x site” were tested for their influence in the composition of *Ae*. *albopictus* microbiome.
(XLSX)

S8 Table Data table for manuscript analysis. Includes OTU counts, site, month, environmental variables (temperature, rainfall, and elevation) for each site, mosquito sex, *Wolbachia* sp., *Asaia* sp., and *Ascogregarina* sp. Ct average values, and *Ascogregarina* sp. presence-absence.
(XLSX)
