# Supplementary material for: The Aedes albopictus (Diptera: Culicidae) microbiome varies spatially and with Ascogregarine infection
Source: PLoS Negl Trop Dis. 2020 Aug 19;14(8):e0008615. doi: 10.1371/journal.pntd.0008615 (PMC7437863; doi:10.1371/journal.pntd.0008615)
Supplement: S1 Text — (DOCX) [file pntd.0008615.s001.docx]

S1 Text. Investigating random interactions to assess overall changes in the composition of the mosquito microbiome

Individual level effects (i.e. Ascogregarina presence and sex) and spatial variation (i.e. Site) were assessed as random interactions in a mixed effects model to test for changes in the mosquito microbiome. The random interactions of “site x Ascogregarina presence” (p=0.99), “OTU x Ascogregarina presence x sex” (p=1), and “OTU x Ascogregarina presence x site” (p=0.22) were not significantly associated with *Ae. albopictus* microbiome (S7 Table).
